# Prevalence of Dementia Among US Adults With Autism Spectrum Disorder

**DOI:** 10.1001/jamanetworkopen.2024.53691

**Published:** 2025-01-02

**Authors:** Giacomo Vivanti, Wei-Lin Lee, Jonas Ventimiglia, Sha Tao, Kristen Lyall, Lindsay L. Shea

**Affiliations:** 1 A.J. Drexel Autism Institute, Drexel University, Philadelphia, Pennsylvania

## Abstract

This cohort study evaluates the prevalence of dementia diagnoses among US adults with autism spectrum disorder.

## Introduction

Studies on the prevalence of dementia in individuals diagnosed with autism spectrum disorder (ASD) have yielded mixed findings, with small sample sizes precluding conclusive inference. One strategy to circumvent this issue is the examination of the nationwide prevalence of dementia among enrollees in public insurance, relied upon by diverse groups of more than 25 million (Medicaid enrollees) and 65 million (Medicare enrollees) older Americans in 2021. Research on Medicaid enrollees^1^ indicated a higher dementia prevalence in enrollees with ASD compared with other Medicaid beneficiaries. A Medicare claims study^2^ documented a 4-fold increase of cognitive disorders diagnoses in beneficiaries with ASD compared with general Medicare population estimates. A limitation to date is the lack of research including linked Medicare and Medicaid data.

## Methods

Following the Strengthening the Reporting of Observational Studies in Epidemiology (STROBE) reporting guidelines, this retrospective cohort study reports the nationwide prevalence of identified dementia diagnoses in individuals with assigned ASD diagnoses in linked Medicare and Medicaid data. The study was approved by the Drexel University institutional review board, which granted a waiver of informed consent due to minimal risk to study subjects and because the research could not practicably be completed without a waiver of consent. Participants were individually linked Medicaid and fee-for-service Medicare beneficiaries aged 30 years and older enrolled January 1, 2014, to December 31, 2016, whose diagnosis of ASD was detected via *International Classification of Diseases, Ninth Revision* or *Tenth Revision* diagnostic codes (eMethods in Supplement 1). Race and ethnicity from Medicare and Medicaid enrollment data gathered by self-report and reporting from others involved in Medicaid enrollment processes were included. Racial and ethnic differences were assessed to track disparities from previous reports and across diagnostic groups. Given the known association between intellectual disabilities (ID) and dementia, we created an ASD-only group including 46 877 individuals and an ASD plus ID group (see eTable in Supplement 1 for ID diagnostic codes) including 67 705 individuals. Following previous research, individuals with Down syndrome were excluded because of their genetic risk for dementia. Diagnosis of dementia was based on the Bynum-standard algorithm,^3^ and validated algorithms from the Chronic Condition Warehouse were applied for all other conditions. Prevalence of dementia diagnoses in each group was examined as a period prevalence estimate over the 3 years of enrollment and annually. Denominators were calculated using the total enrolled for each group. Additionally, we examined whether common risk factors for dementia increased the odds of assigned dementia diagnoses in the sample. Data were analyzed from June 2023 to June 2024.

## Results

Of the 114 582 individuals included in the study, most (63 194 [55.2%]) were aged 40 or older, 80 104 (69.9%) were male, 18 627 (16.3%) were Black, 8048 (7.02%) were Hispanic or Latino, and 80 680 (70.4%) were White. An identified dementia diagnosis was present in 8.03% of the ASD-only group and 8.88% of the ASD plus ID group (Table 1). The odds of a dementia diagnosis increased with age (Table 2), with a prevalence of 35.12% in the ASD-only group and 31.22% in the ASD plus ID group for individuals older than 64. The odds were higher in individuals with cardiovascular risk factors and depression or other psychiatric conditions, after controlling for residence state (Table 2).

**Table 1.** Characteristics of the 2 Groups of Interest Stratified According to Demographics and Features Known to Be Associated With Risk of Dementia

Characteristics	Participants, No. (%)			
	ASD only		ASD plus ID	
	No dementia (n = 43 111)	With dementia (n = 3766)	No dementia (n = 61 691)	With dementia (n = 6014)
Age group, y				
30-39	20 754 (48.14)	655 (17.39)	28 616 (46.39)	1363 (22.66)
40-49	9593 (22.25)	407 (10.81)	14 800 (23.99)	1105 (18.37)
50-59	7209 (16.72)	609 (16.17)	11 824 (19.17)	1453 (24.16)
60-64	2288 (5.31)	326 (8.66)	3533 (5.73)	768 (12.77)
≥65	3267 (7.58)	1769 (46.97)	2918 (4.73)	1325 (22.03)
Sex				
Male	30 969 (71.84)	2284 (60.65)	42 870 (69.49)	3981 (66.20)
Female	12 142 (28.16)	1482 (39.35)	18 821 (30.51)	2033 (33.80)
Race and ethnicity^a^				
American Indian/Alaska Native	290 (0.67)	NR^b^	348 (0.56)	NR^b^
Asian/Pacific Islander	1042 (2.42)	80 (2.12)	1623 (2.63)	100 (1.66)
Black	5041 (11.69)	486 (12.90)	12 095 (19.61)	1005 (16.71)
Hispanic/Latino	2714 (6.30)	219 (5.82)	4768 (7.73)	347 (5.77)
>1 Category selected	43 (0.10)	NR^b^	89 (0.14)	NR^b^
White	32 478 (75.34)	2833 (75.23)	40 956 (66.39)	4413 (73.38)
Missing	1503 (3.48)	121 (3.21)	1812 (2.94)	118 (1.96)
Urbanicity				
Urban	27 961 (64.86)	2692 (71.48)	36 518 (59.20)	4093 (68.06)
Suburban	7913 (18.35)	615 (16.33)	9739 (15.79)	908 (15.10)
Rural	1588 (3.68)	133 (3.53)	1407 (2.28)	124 (2.06)
Missing	5649 (13.10)	326 (8.66)	14 027 (22.74)	889 (14.78)
Diagnoses				
Depression	16 296 (37.80)	2142 (56.88)	14 447 (23.42)	2635 (43.81)
Other mental health	29 085 (67.47)	3163 (83.99)	44 517 (72.16)	5489 (91.27)
Cardiovascular disease	21 702 (50.34)	2936 (77.96)	29 830 (48.35)	4275 (71.08)

Abbreviations: ASD, autism spectrum disorder; ID, intellectual disability; NR, not reported.

^a^ Participant race is based on self-reported information by beneficiaries at the time of Medicaid/Medicare enrollment.

^b^ Data were suppressed in these cells as required by the Centers for Medicare and Medicaid Services.

**Table 2.** Odds Ratios (ORs) of Dementia in the 3 Groups According to Age Group, Sex, Race, Urbanicity, and Presence or Absence of Diagnoses Including Depression, Other Psychiatric Conditions, and Cardiovascular Disease Risk Factors, Controlling for State of Enrollment

Characteristic	OR (95% CI)	
	ASD only	ASD plus ID
Age group, y		
30-39	1 [Reference]	1 [Reference]
40-49	1.27 (1.11-1.44)	1.46 (1.34-1.59)
50-59	2.48 (2.19-2.8)	2.24 (2.07-2.44)
60-64	4.17 (3.59-4.84)	4.02 (3.63-4.46)
≥65	18.15 (16.25-20.27)	8.64 (7.87-9.48)
Female sex	1.23 (1.14-1.33)	1.07 (1.00-1.13)
Race and ethnicity^a^		
American Indian/Alaska Native	1.39 (0.87-2.21)	0.96 (0.63-1.46)
Asian/Pacific Islander	1.49 (1.14-1.94)	0.95 (0.75-1.19)
Black	1.37 (1.22-1.55)	0.87 (0.80-0.94)
Hispanic/Latino	1.22 (1.04-1.44)	0.96 (0.84-1.08)
Missing	0.95 (0.77-1.18)	0.80 (0.65-0.98)
>1 Category selected	1.01 (0.21-4.75)	0.59 (0.18-1.91)
White	1 [Reference]	1 [Reference]
Urbanicity		
Rural	1 [Reference]	1 [Reference]
Suburban	1.03 (0.82-1.29)	1.01 (0.81-1.24)
Urban	1.08 (0.87-1.34)	1.11 (0.91-1.37)
Diagnoses		
Depression	2.55 (2.29-2.82)	2.74 (2.49-3.02)
Other mental health	1.60 (1.48-1.74)	1.86 (1.75-1.98)
Cardiovascular disease	1.78 (1.63-1.95)	1.70 (1.60-1.82)

Abbreviations: ASD, autism spectrum disorder; ID, intellectual disability.

^a^ Participant race is based on self-reported information by beneficiaries at the time of Medicaid/Medicare enrollment.

## Discussion

Linked Medicaid and Medicare records suggest a markedly elevated prevalence of identified dementia diagnoses in individuals with an ASD diagnosis. Findings were consistent with our previous research,^1^ and prevalence estimates were higher than those for the general population of Medicaid and Medicare beneficiaries reported in the literature.^1,4^

Although the scale of this study is, to our knowledge, unprecedented for this area of research, findings should be considered as hypothesis-generating data to be substantiated by further research. ASD and dementia are diagnostic constructs encompassing different conditions—future research should focus on groups defined by cause. Additionally, there was no comparison group or clinical assessment for diagnoses. However, the correspondence between case identification via claims data and clinical diagnoses of ASD and dementia in previous research approaches 90%.^5,6^ The use of datasets dating back almost a decade and the circumscribed focus on insurance beneficiaries are further limitations.

Our data highlight the importance of health policy efforts for the growing ASD population at risk for or affected by dementia. Future research should address factors that might contribute to the cooccurrence of neurodevelopmental and neurodegenerative conditions, including barriers to accessing educational and social opportunities, as well as biological mechanisms of shared pathophysiology.

## Notes

Supplement 1. **eMethods. **Data Extraction and Group Operationalization **eTable. **ICD Codes For All Variables

Supplement 2.
Data Sharing Statement
