# Error in Funding/Support Section

**DOI:** 10.1001/jamanetworkopen.2025.8224

**Published:** 2025-03-31

In the Research Letter titled “Prevalence of Dementia Among US Adults With Autism Spectrum Disorder,”^1^ published January 2, 2025, there was an error in the Funding/Support section. The text should have indicated that grant No. R01NS131433 was awarded to Brian K. Lee, PhD. An acknowledgment of Dr Lee's contribution has also been added to the Article Information. This article has been corrected.^1^
