# Supplementary material for: Prevalence of Dementia Among US Adults With Autism Spectrum Disorder
Source: JAMA Netw Open. 2025 Jan 2;8(1):e2453691. doi: 10.1001/jamanetworkopen.2024.53691 (PMC11696448; doi:10.1001/jamanetworkopen.2024.53691)
Supplement: Supplement 1. — eMethods. Data Extraction and Group Operationalization eTable. ICD Codes For All Variables [file jamanetwopen-e2453691-s001.pdf]

Supplemental Online Content

Vivanti G, Lee WL, Ventimiglia J, Tao S, Lyall K, Shea LL. Prevalence of dementia among US adults with autism spectrum disorder. *JAMA Netw Open*. 2024;8(1):e2453691. doi:10.1001/jamanetworkopen.2024.53691

eMethods. Data Extraction and Group Operationalization
eTable. ICD Codes For All Variables

This supplemental material has been provided by the authors to give readers additional information about their work.

© 2025 Vivanti G et al. *JAMA Network Open*.

eMethods. Data Extraction and Group Operationalization

Medicaid data files included the national Medicaid Analytics eXtract files (MAX) and the T-MSIS Analytic Files (TAF). Medicare data files include Part A and B. Data across Medicaid and Medicare include individual demographic information and eligibility classification in a beneficiary summary file, as well as service files for inpatient, other outpatient/ambulatory therapy, long-term care, and prescription drug claims that contain information on diagnoses, treatments, and reimbursement. A unique identifier allows linking of multiple claims for an individual over time.

Data were extracted for the following two diagnostic categories of interest, further characterized in Table 1.

ASD only group. Inclusion in the ASD only diagnostic group required 2 outpatient or 1 inpatient claim with a diagnosis associated with ASD (ICD-9 codes 299.xx or ICD-10 F84.x) and with no co-occurring intellectual disability diagnosis (ICD-9 codes 317.xx – 319.xx).

ASD+ID group. The ASD+ID group was based on the same ASD requirement as the ASD only group, but also had a co-occurring ID diagnosis (2 outpatient claims or 1 inpatient claim associated with an ID diagnosis of 317.xx – 319.xx) during the study period. Individuals with Down syndrome were excluded due to the high prevalence of dementia in this population.

Individuals across diagnostic groups were required to have at least 9 out of 12 months of Medicaid or Medicare enrollment between January 1, 2014, and December 31, 2015, to account for administrative churning, or abbreviated disruptions in Medicaid enrollment due to administrative processes. We followed them from the date of meeting inclusion criteria until a loss of Medicaid or Medicare eligibility, a date of death, or December 31, 2016.

© 2025 Vivanti G et al. *JAMA Network Open.*

eTable. ICD Codes For All Variables

Diagnosis	Requirement	ICD-9
Autism Spectrum Disorder (ASD)	1 IP or 2 LT/OT	99.0, 299.00, 299.01, 299.1, 299.11, 299.8, 299.80, 299.81, 299.9, 299.90, 299.91
Intellectual Disabilities (ID)	1 IP or 2 LT/OT	317, 318, 318.0, 318.1, 318.2, 319
Alzheimer's Disease and Related Disorders or Senile Dementia (Bynum)	1 Claim	"3310", "33111", "33119", "3312", "3317", "33182", "33189", "2900", "29010", "29011", "29012", "29013", "29020", "29021", "2903", "29040", "29041", "29042", "29043", "2908", "2940", "29410", "29411", "29420", "29421", "2948", "797", "F0150", "F0151", "F0280", "F0281", "F0390", "F0391", "F04", "G138", "F05", "F061", "F068", "G300", "G301", "G308", "G309", "G311", "G312", "G3101", "G3109", "G3183", "G3189", "G94", "R4181", "R54"
Depression	1 Claim	"29620", "29621", "29622", "29623", "29624", "29625", "29626", "29630", "29631", "29632", "29633", "29634", "29635", "29636", "29651" , "29652", "29653", "29654", "29655", "29656", "29660", "29661", "29662", "29663", "29664", "29665", "29666", "29689", "3004", "311", "2980", "3091", "F320", "F321", "F322", "F323", "F324", "F325", "F3289", "F329", "F32A", "F330", "F331", "F332", "F333", "F3340", "F3341", "F3342", "F338", "F339", "F341"
Other Mental Health Disorder	1 Claim	290.XX - 320.XX, and all F code other than the ones listed in ASD, ID, Dementia and Depression<br><br>**Not to include:**<br>**ASD:** '299', '2990', '29900', '29901', '2991', '29910', '29911', '2998', '29980', '29981', '2999', '29990', '29991', 'F84', 'F840', 'F842', 'F843', 'F845', 'F848', 'F849', '317', '318', '3180', '3181', '3182', '319', 'F70', 'F71',<br>**ID:** 'F72', 'F73', 'F78', 'F78A', 'F78A1', 'F78A9', 'F79'<br>**ADRD:** "2900", "29010", "29011", "29012", "29013", "29020", "29021", "2903", "29040", "29041", "29042", "29043", "2940", "29410", "29411", "29420", "29421", "2948", "797","F0150", "F0151", "F0280", "F0281", "F0390", "F0391", "F04", "F05", "F061", "F068"<br>**Depression:** "29620", "29621", "29622", "29623", "29624", "29625", "29626", "29630", "29631", "29632",

© 2025 Vivanti G et al. *JAMA Network Open.*

		"29633", "29634", "29635", "29636", "29651" , "29652", "29653", "29654", "29655", "29656", "29660", "29661", "29662", "29663", "29664", "29665", "29666", "29689", "3004", "311", "2980", "3091", "F320", "F321", "F322", "F323", "F324", "F325", "F3289", "F329", "F32A", "F330", "F331", "F332", "F333", "F3340", "F3341", "F3342", "F338", "F339", "F341"
Cardiovascular Disease Risk Factor		Any of the following 3 diagnosis
Hypertension	1 IP or 2 LT/OT	"36211", "4010", "4011", "4019", "40200", "40201", "40210", "40211", "40290", "40291", "40300", "40301", "40310", "40311", "40390", "40391", "40400", "40401", "40402", "40403", "40410", "40411", "40412", "40413", "40490", "40491", "40492", "40493", "40501", "40509", "40511", "40519", "40591", "40599", "4372", "H35031", "H35032", "H35033", "H35039", "I10", "I110", "I119", "I120", "I129", "I130", "I1310", "I1311", "I132", "I150", "I151", "I152", "I158", "I159", "I674", "N262"
Obesity	1 IP or 2 LT/OT	"2780", "27800", "27801", "27803", "V853", "V8530", "V8531", "V8532", "V8533", "V8534", "V8535", "V8536", "V8537", "V8538", "V8539", "V854", "V8541", "V8542", "V8543", "V8544", "V8545", "E6601", "E6609", "E661", "E662", "E668", "E669", "Z6830", "Z6831", "Z6832", "Z6833", "Z6834", "Z6835", "Z6836", "Z6837", "Z6838", "Z6839", "Z6841", "Z6842", "Z6843", "Z6844", "Z6845"

Diabetes	1 IP or 2 LT/OT	"24900", "24901", "24910", "24911", "24920", "24921", "24930", "24931", "24940", "24941", "24950", "24951", "24960", "24961", "24970", "24971", "24980", "24981", "24990", "24991", "25000", "25001", "25002", "25003", "25010", "25011", "25012", "25013", "25020", "25021", "25022", "25023", "25030", "25031", "25032", "25033", "25040", "25041", "25042", "25043", "25050", "25051", "25052", "25053", "25060", "25061", "25062", "25063", "25070", "25071", "25072", "25073", "25080", "25081", "25082", "25083", "25090", "25091", "25092", "25093", "3572", "36201", "36202", "36203", "36204", "36205", "36206", "36641", "E0800", "E0801", "E0810", "E0811", "E0821", "E0822", "E0829", "E08311", "E08319", "E08321", "E083211", "E083212", "E083213", "E083219", "E08329", "E083291", "E083292", "E083293", "E083299", "E08331", "E083311", "E083312", "E083313", "E083319", "E08339", "E083391", "E083392", "E083393", "E083399", "E08341", "E083411", "E083412", "E083413", "E083419", "E08349", "E083491", "E083492", "E083493", "E083499", "E08351", "E083511", "E083512", "E083513", "E083519", "E083521", "E083522", "E083523", "E083529", "E083531", "E083532", "E083533", "E083539", "E083541", "E083542", "E083543", "E083549", "E083551", "E083552", "E083553", "E083559", "E08359", "E083591", "E083592", "E083593", "E083599", "E0836", "E0837X1", "E0837X2", "E0837X3", "E0837X9", "E0839", "E0840", "E0841", "E0842", "E0843", "E0844", "E0849", "E0851", "E0852", "E0859", "E08610", "E08618", "E08620", "E08621", "E08622", "E08628", "E08630", "E08638", "E08641", "E08649", "E0865", "E0869", "E088", "E089", "E0900", "E0901", "E0910", "E0911", "E0921", "E0922", "E0929", "E09311", "E09319", "E09321", "E093211", "E093212", "E093213", "E093219", "E09329", "E093291", "E093292", "E093293", "E093299", "E09331", "E093311",

© 2025 Vivanti G et al. *JAMA Network Open*.

"E093312", "E093313", "E093319", "E09339", "E093391", "E093392", "E093393", "E093399", "E09341", "E093411", "E093412", "E093413", "E093419", "E09349", "E093491", "E093492", "E093493", "E093499", "E09351", "E093511", "E093512", "E093513", "E093519", "E093521", "E093522", "E093523", "E093529", "E093531", "E093532", "E093533", "E093539", "E093541", "E093542", "E093543", "E093549", "E093551", "E093552", "E093553", "E093559", "E09359", "E093591", "E093592", "E093593", "E093599", "E0936", "E0937X1", "E0937X2", "E0937X3", "E0937X9", "E0939", "E0940", "E0941", "E0942", "E0943", "E0944", "E0949", "E0951", "E0952", "E0959", "E09610", "E09618", "E09620", "E09621", "E09622", "E09628", "E09630", "E09638", "E09641", "E09649", "E0965", "E0969", "E098", "E099", "E1010", "E1011", "E1021", "E1022", "E1029", "E10311", "E10319", "E10321", "E103211", "E103212", "E103213", "E103219", "E10329", "E103291", "E103292", "E103293", "E103299", "E10331", "E103311", "E103312", "E103313", "E103319", "E10339", "E103391", "E103392", "E103393", "E103399", "E10341", "E103411", "E103412", "E103413", "E103419", "E10349", "E103491", "E103492", "E103493", "E103499", "E10351", "E103511", "E103512", "E103513", "E103519", "E103521", "E103522", "E103523", "E103529", "E103531", "E103532", "E103533", "E103539", "E103541", "E103542", "E103543", "E103549", "E103551", "E103552", "E103553", "E103559", "E10359", "E103591", "E103592", "E103593", "E103599", "E1036", "E1037X1", "E1037X2", "E1037X3", "E1037X9", "E1039", "E1040", "E1041", "E1042", "E1043", "E1044", "E1049", "E1051", "E1052", "E1059", "E10610", "E10618", "E10620", "E10621", "E10622", "E10628", "E10630", "E10638", "E10641", "E10649", "E1065", "E1069", "E108", "E109", "E1100", "E1101", "E1110", "E1111", "E1121", "E1122", "E1129",

"E11311", "E11319", "E11321", "E113211", "E113212", "E113213", "E113219", "E11329", "E113291", "E113292", "E113293", "E113299", "E11331", "E113311", "E113312", "E113313", "E113319", "E11339", "E113391", "E113392", "E113393", "E113399", "E11341", "E113411", "E113412", "E113413", "E113419", "E11349", "E113491", "E113492", "E113493", "E113499", "E11351", "E113511", "E113512", "E113513", "E113519", "E113521", "E113522", "E113523", "E113529", "E113531", "E113532", "E113533", "E113539", "E113541", "E113542", "E113543", "E113549", "E113551", "E113552", "E113553", "E113559", "E11359", "E113591", "E113592", "E113593", "E113599", "E1136", "E1137X1", "E1137X2", "E1137X3", "E1137X9", "E1139", "E1140", "E1141", "E1142", "E1143", "E1144", "E1149", "E1151", "E1152", "E1159", "E11610", "E11618", "E11620", "E11621", "E11622", "E11628", "E11630", "E11638", "E11641", "E11649", "E1165", "E1169", "E118", "E119", "E1300", "E1301", "E1310", "E1311", "E1321", "E1322", "E1329", "E13311", "E13319", "E13321", "E133211", "E133212", "E133213", "E133219", "E13329", "E133291", "E133292", "E133293", "E133299", "E13331", "E133311", "E133312", "E133313", "E133319", "E13339", "E133391", "E133392", "E133393", "E133399", "E13341", "E133411", "E133412", "E133413", "E133419", "E13349", "E133491", "E133492", "E133493", "E133499", "E13351", "E133511", "E133512", "E133513", "E133519", "E133521", "E133522", "E133523", "E133529", "E133531", "E133532", "E133533", "E133539", "E133541", "E133542", "E133543", "E133549", "E133551", "E133552", "E133553", "E133559", "E13359", "E133591", "E133592", "E133593", "E133599", "E1336", "E1339", "E1340", "E1341", "E1342", "E1343", "E1344", "E1349", "E1351", "E1352", "E1359", "E13610", "E13618", "E13620", "E13621", "E13622", "E13628", "E13630", "E13638",

© 2025 Vivanti G et al. *JAMA Network Open*.

"E13641", "E13649", "E1365", "E1369", "E138", "E139"

© 2025 Vivanti G et al. *JAMA Network Open*.
