# Supplementary material for: Prevalence of Dementia Among US Adults With Autism Spectrum Disorder
Source: JAMA Netw Open. 2025 Jan 2;8(1):e2453691. doi: 10.1001/jamanetworkopen.2024.53691 (PMC11696448; doi:10.1001/jamanetworkopen.2024.53691)
Supplement: Supplement 2. — Data Sharing Statement [file jamanetwopen-e2453691-s002.pdf]

# Data Sharing Statement

Vivanti. Prevalence of Dementia Among US Adults With Autism Spectrum Disorder. *JAMA Netw Open*. Published January 02, 2025. doi:10.1001/jamanetworkopen.2024.53691

**Data**
**Data available:** No

**Additional Information**
**Explanation for why data not available:** Centers for Medicare and Medicaid (CMS) governs the use of Medicare and Medicaid claims data and does not permit the distribution of individual-level data without a data use agreement directly with CMS.
